# A Pilot Feasibility Study of a Home Tablet-Based Neurorehabilitation Program and Serial Brain Vital Sign Monitoring for Survivors of Pediatric Cerebral Malaria

**DOI:** 10.4269/ajtmh.25-0492

**Published:** 2025-12-16

**Authors:** Sarah J. Macoun, Jessica M. Silverman, Philippe R. Brunet, Jessica M. Lewis, Edith Kafoteka, Thereza Ziba, Eric D. Kirby, Joshua Ighalo, Ryan C. N. D’Arcy, Nicole O’Brien

**Affiliations:** ^1^ Child Neuropsychology Lab, University of Victoria, Victoria, British Columbia, Canada;; ^2^ The Blantyre Malaria Project, Queen Elizabeth Central Hospital, Blantyre, Malawi;; ^3^ Centre for Neurology Studies, HealthTech Connex, Metro Vancouver, British Columbia, Canada;; ^4^ BrainNET, Faculty of Applied Sciences, Simon Fraser University, Metro Vancouver, British Columbia, Canada;; ^5^ DM Centre for Brain Health, Radiology, University of British Columbia, Metro Vancouver, British Columbia, Canada;; ^6^ Nationwide Children’s Hospital/The Ohio State University, Columbus, Ohio

## Abstract

Lasting sequelae are identified in 50% of child survivors of cerebral malaria (CM). Rehabilitation options in malaria-endemic regions are scarce and largely focused on physical deficits, leaving children without support for cognitive recovery. Effective and accessible interventions are vital for improving outcomes. Furthermore, the assessment of brain function and recovery after CM is dependent on behavior-based tests that are time-consuming and require substantial training to administer. Objective, easy-to-use alternatives may be impactful. Children aged 3–12 years who had survived CM were recruited. Participants underwent a 6-month in-home, tablet-based neurorehabilitation program called Dino Island (DI). Participants also underwent serial assessments of brain health that were conducted by measuring event-related potentials (ERPs) using the Brain Vital Signs system. The feasibility, fidelity, acceptability, appropriateness, and affordability of the interventions were evaluated through interviews with the study nurses and the participants’ families. Both programs were feasible and easy to implement. Acceptability was demonstrated by low attrition rates (5%) and positive family ratings (100%). Appropriateness for DI was confirmed by parent reports of positive behavioral changes in their children (60%). For Brain Vital Signs, appropriateness was confirmed by adequate data acquisition for most participants. Finally, positive indicators of affordability from a healthcare perspective were identified. Neurorehabilitation using a home tablet-based program and objective brain health assessment using ERPs was feasible, well accepted, and appropriate in child CM survivors in sub-Saharan Africa. Further development and research into the program’s ability to improve and measure cognitive recovery is justified.

## INTRODUCTION

Malaria affects 249 million people annually, causing 608,000 deaths.^1^ Cerebral malaria (CM) is a severe form of the disease that affects 575,000 people globally, with 90% of cases occurring in children under 5 years of age.^2^^–^^4^ Because of advances in medical treatment, ∼80% of children now survive CM.^5^^–^^8^ However, 50% of survivors experience long-lasting neurologic sequelae, making CM the leading cause of pediatric neuro-disability in sub-Saharan Africa.^9^^–^^12^ Cognitive deficits in attention and executive functions (A/EF; e.g., working memory, inhibitory control, self-regulation, and cognitive flexibility) occur frequently in CM survivors.^13^^–^^17^ These deficits negatively affect families both socially and economically.^18^^,^^19^

Unfortunately, because of a lack of therapists and logistical issues, families in sub-Saharan Africa experience significant barriers in accessing rehabilitation services for their children.^20^^–^^22^ Previous research on hospital- or school-based computerized cognitive rehabilitation therapy (CCRT) in sub-Saharan Africa clearly indicates efficacy in improving cognitive function in children with neurodisability due to various etiologies, including HIV and CM.^23^^–^^32^ However, the generalizability of this approach may be limited by the need for families to travel regularly to the center where CCRT takes place or for personnel to travel to the child’s school with the necessary equipment to complete a session. Home-based, parent-delivered computerized neuro-rehabilitation programs could be an effective option with improved accessibility for children with CM.^33^^–^^36^ However, research on the feasibility of this type of intervention in this population is limited.

Furthermore, assessing a child’s neurocognitive recovery is difficult in the African context because many countries lack the trained personnel needed to perform comprehensive neurocognitive assessments. Additionally, these outcome measures are often behavior-based, depending heavily on the patient’s desire or capacity to participate in testing and can therefore be error-prone. One study has revealed that measuring event-related potentials (ERPs; electrical signals generated in the brain in response to a specific stimulus) may be an alternative, objective means of assessing cognitive function in African children.^37^ Further evaluations of the utility of ERP assessment in this context have not been conducted.

**Figure 1.**
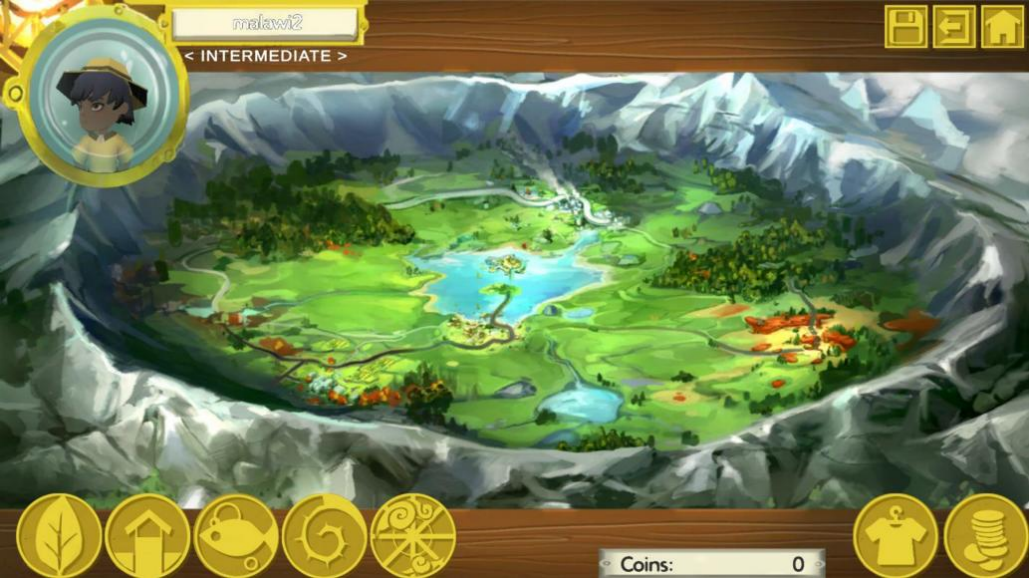
Dino Island world map, with personalized avatar. The icons at the bottom represent entry into each of the five therapeutic games and the Dino Island motivation system.

**Figure 2.**
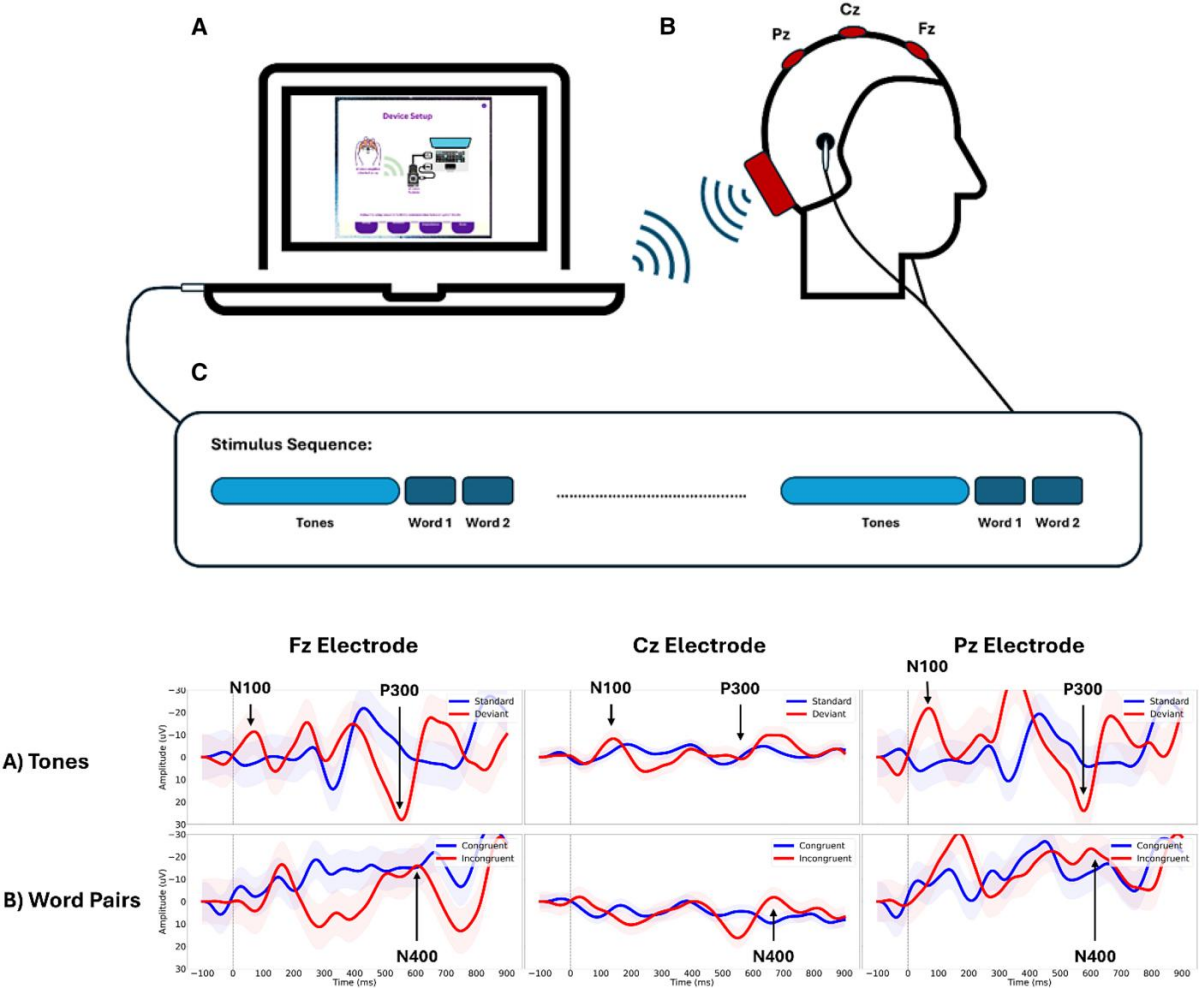
The Brain Vital Signs framework integrates auditory tone stimuli and word pair primes to elicit the N100, P300, and N400 event-related potential (ERP) responses. Top: Overall schematic of the electroencephalography recording (right), which is wirelessly connected to the laptop to deliver the auditory stimuli and record the ERP data. Bottom: Brain vital sign ERP responses for the tone stimuli. (**A**) Responses to standard tones are shown in blue, and deviants are shown in red. The N100 and P300 responses are indicated at all three electrode sites. (**B**) Word pair stimuli: Responses to congruent word pairs (e.g., bread–butter) are shown in blue, and incongruent word pairs (e.g., bread–donkey) are shown in red. N400 responses are indicated in all three electrode sites. Time (ms) is shown on the *x*-axis, and voltage (microvolts) is shown on the *y*-axis (negative is plotted up).

Therefore, two novel technologies were piloted in child survivors of CM in Malawi, Africa. The first of these, Dino Island (DI), is a computerized, tablet-based neurocognitive rehabilitation program delivered at home.^38^^–^^40^ Grounded in principles of experience-dependent neuroplasticity, DI employs a hierarchical, adaptive framework of repetitive, process-specific cognitive exercises targeting key A/EF abilities, such as inhibitory control, focused attention, sustained attention, working memory, and cognitive flexibility, in both the visual and auditory domains.^41^^–^^46^ Specifically, DI consists of five adaptive, increasingly difficult therapeutic games targeting different aspects of A/EF, paired with parent-trained metacognitive coaching (Figure 1).^47^^–^^52^ As the child gains proficiency, each game progressively increases in difficulty by modifying level duration, distractions, rules, and instructions, thereby systematically training both basic and higher-order A/EF processes. Dino Island is a hybrid intervention that combines process-specific training with compensatory training, in line with best practice recommendations for meaningful and generalizable gains.^39^^,^^41^^,^^45^ The second technology, Brain Vital Signs, measures ERPs to objectively assess a spectrum of brain functioning. Specifically, neural responses for auditory sensation (N100 response), basic attention (P300 response), and cognitive processing (N400 response) are measured (Figure 2).^53^^–^^57^

## MATERIALS AND METHODS

### Participants.

Children were recruited at Queen Elizabeth Central Hospital (QECH) through the Blantyre Malaria Project in Blantyre, Malawi. Eligible participants were CM survivors aged 3–12 years who had a caregiver willing and able to complete DI program training and deliver DI at home and were willing and able to undergo the Brain Vital Signs assessments at follow-up visits 1, 3, and 6 months after hospital discharge. Children were ineligible for participation if they lacked the sensory or motor functions necessary to engage with the tablet-based intervention. Families of eligible children provided written consent before participating.

### Implementation procedures.

#### Stage 1: Implementation planning.

A multi-disciplinary implementation team, including members both on-site (two neurodevelopmental nurses, a physician, and parents of CM survivors) and off-site (a pediatric neuropsychologist, a cognitive neuroscientist, and their research teams), was assembled.^58^ The on-site team conducted a needs assessment and evaluated the available supports for deploying the proposed program in Malawi. The core components of DI and Brain Vital Signs were reviewed by the off-site team to identify required cultural and clinical adaptations. The core elements comprising DI when used in other settings include 1) a parent or adult coach trained to deliver the DI intervention (typically 2–3 hours of training), who monitors 100% of the child’s gameplay and teaches and facilitates the child’s use of metacognitive strategies during subsequent uses, and 2) DI game play for a minimum target of 15 hours, distributed over 6–8 weeks (2–4 times/week), with equal distribution across DI’s five therapeutic games. Decisions regarding intervention duration and intensity are made on the basis of similar or related research in child populations and best-practice guidelines.^40^^,^^41^^,^^50^^,^^59^

Adaptations made to the DI program for the Malawian context are as follows: 1) a train-the-trainer model was used, whereby the DI research team trained nurses to teach parents how to use DI, 2) verbal instructions were given in Chichewa to parents during each DI training for each game, 3) additional training instructions were provided to parents on the use of tablets and solar charging technologies (tablets were locked with administrator privileges to reduce complexity and limit their use to the Dino Island application), 4) visuals were integrated to help address literacy and language barriers, and 5) the timeline of DI was extended to accommodate treatment plans for CM. Dino Island sessions were expected to total 1.5 hours per week for 6 months, while avoiding intervention gaps longer than 1 week between sessions.

Modifications made to the Brain Vital Signs program are as follows: 1) translation of the auditory stimuli into Chichewa, 2) modification of the data-transfer protocol, and 3) provision of remote video-based training to nurses on program utilization.

#### Stage 2: Program delivery.

Ten Android tablets (Samsung, Suwon-si, South Korea), solar panels (Kohree 100W [Kohree, Charlotte, NC] or Togo 120W [Togo Power Inc., Walnut, CA]), charging cables, portable power banks (Eteilymon 20,000 mAh [Paey Intelligence (Shenzhen) Technology Co., Ltd. China]), and earphones (Geekria Children’s in-Ear [Geekria, Cupertino, CA]) were purchased and shipped to Malawi; each family left the hospital with one complete set of equipment required for executing the DI program at home. Nurse–family training for DI took place during the child’s hospital stay, lasting 30–45 minutes daily over 3 days, and focused on critical program components, including the delivery of DI games and how parents or guardians should act as metacognitive coaches. Nurses reviewed training materials with parents, modeled the game’s delivery, and then observed parents delivering the game to their child to ensure adequate understanding and skill. Participants were discharged from the hospital only when a clear ability to use the program was demonstrated. Nurses provided extra support to families after discharge via telephone calls and home visits, as needed.

Brain Vital Signs were assessed at hospital discharge and at each scheduled follow-up visit (1, 3, and 6 months after admission). Electroencephalogram (EEG) data were collected using an eight-channel Nautilus EEG system (g.tec Medical Engineering, Schiedlberg, Austria), with an elasticized cloth cap containing an EEG amplifier and standardized electrode locations (Fz, Cz, and Pz). Three additional electrodes included electrooculogram electrodes (vertical electrooculogram and horizontal electrooculogram) and ground and reference electrodes (right earlobe). Impedances were below 20 KOhms. All scans were conducted in a quiet, closed room. To reduce motor and ocular artifacts, participants were instructed to sit motionlessly and maintain visual fixation on a cross positioned at eye level, 2 meters away.

The off-site DI and Brain Vital Signs research teams provided ongoing consultation, troubleshooting, and real-time adaptations to the programs as barriers were identified by the on-site implementation team.

#### Stage 3: Outcomes.

The following five implementation outcomes were analyzed via semi-structured interviews with parents at all follow-up visits, as well as with study nurses at the completion of years 1 and 2 of the program:

1) Feasibility: Feasibility refers to the ease of intervention and assessment delivery within the setting and was assessed by evaluating barriers to delivery (equipment, time, geography, and tablet usability).
2) Fidelity: Fidelity refers to the intervention being delivered as intended and was measured using program compliance and completion rates.
3) Acceptability: Acceptability refers to community willingness and perceptions of success, including stigma, support, and resource adequacy, and was assessed through exit interviews with families and the hospital team.
4) Appropriateness: Appropriateness refers to the evaluation of the fit of DI and Brain Vital Signs in the Malawian context through their impact on cognitive and behavioral outcomes. It was assessed through qualitative exit interviews with families and the hospital team.
5) Affordability: Affordability refers to the assessment of costs to families and overall impact on the health system, which was evaluated through exit interviews with families and the hospital team.^60^^,^^61^

Nurses conducted in-person interviews with parents in Chichewa, which they later transcribed into English. The research team conducted interviews with the nurses in English via Zoom (Zoom Communications, Inc., San Jose, CA). Interviews were transcribed and analyzed in NVivo 14 (Lumivero, Denver, CO) using qualitative deductive thematic analysis.^62^^,^^63^ The analysis involved 1) open coding, which involves clustering and organizing participant responses across all three time points, 2) axial coding, which involves grouping questions by core concepts and aligning them with implementation outcomes, and 3) selective coding, which involves comparing codes among participants to identify emerging secondary themes.

## RESULTS

Of the first 22 eligible children, two families declined participation because of concerns about keeping equipment safe in their homes. The other 20 families consented to participate; 10 were enrolled in year 1, and 10 were enrolled in year 2. Participants came from 18 different villages, averaging 32 kilometers (±26) from QECH. The mean age of the participants was 5.7 years (±2.5), and nine were male. Clinical parameters for each participant are presented in Table 1. The program had low attrition, with only one family (5%) withdrawing because their child wanted to use the tablet excessively. The remaining participants completed the DI program in its entirety and presented for all follow-up assessments, during which Brain Vital Signs were measured.

**Table 1:** Participant demographics (*N* = 19)

Patient ID	Age	Sex	Village: Distance in Kilometers from QECH	Coma Duration (hours)	Seizures Before Presentation (0: no, 1: yes)	Blantyre Coma Score at Presentation	Time to Normalization of Blantyre Coma Score (hours)	Duration of Hospital Stay (days)
01	7	Male	Pensulo: 11.6	96	1	2	94	6
02	9	Female	Zomba: 66.2	10	1	1	>168	31
03	3	Male	Ndirande: 6.7	15	1	1	96	8
04	3	Female	Thyolo: 39.9	22	1	1	36	6
05	10	Female	Kapichi: 89.6	37	0	1	60	5
06	6	Male	Chiradzulu: 24.6	49	0	2	26	7
07	5	Male	Mwambo: 95.2	48	0	1	32	3
08	8	Female	Chiradzulu: 24.6	48	1	2	34	21
09	5	Male	Chikumba: 64.9	120	1	1	70	8
10	9	Male	Chisempharia: 30	86	1	2	34	4
11	3	Male	Malizani: 10	43	1	2	45	5
12	4	Female	Munzembere: 21.3	56	1	2	28	3
13	4	Female	Mpemba: 13.8	26	0	2	42	4
14	4	Female	Soche Fed: 10	31	1	1	60	7
15	3	Female	Mpemba: 13.8	65	0	0	88	10
16	10	Female	Chatha: 10	12	1	1	45	6
17	6	Female	Tuwaya: 38	14	1	2	19	3
18	4	Male	Mwadzanga: 18.6	18	0	2	31	5
19	4	Female	Makenjira: 16	20	1	2	28	5

QECH = Queen Elizabeth Central Hospital.

Study nurses reported that the adapted DI training model was highly feasible and integrated well into their existing pediatric CM care model. Technological barriers were present for families unfamiliar with using tablets, leading to accidental deletions of user accounts or the DI app during the first month for 100% of the first cohort of families (*n* = 10). This issue was successfully addressed for the second cohort of families by implementing password protection. Nurses provided additional support by phone to 70% of families and conducted nine house visits to address questions or concerns about the program or equipment. Although feasible, these visits imposed an unforeseen time and financial burden due to unplanned travel to participants’ villages.

The program schedule was well-received by most parents, who found the frequency and timing suitable. However, 89% suggested extending the DI gameplay beyond 20-minute sessions, and 44% of families expressed a desire for more game levels, suggesting that expansions may be warranted to support prolonged gameplay. Despite adaptations like vocabulary sheets and nurses training families in Chichewa, language remained a significant challenge, especially for games with complex instructions, leading to reduced play time on these specific games. Trained family members reported being present for 100% of gameplay and successfully using metacognitive strategies with their children. While multiple family members and friends often participated in sessions to support translation, this community approach, although different from North American implementation, did not compromise program fidelity and had unintended social benefits.

Most parents reported notable improvements in memory (66%, 66%, and 100%) and ability to follow instructions (54%, 43%, and 100%), as well as improved mood and behavior (74%, 77%, and 60%), in their child at each of the follow-up time points (1 month, 3 months, and 6 months after hospital discharge). Many parents also reported gains in attention span, emotional regulation, flexibility with transitions, and task completion. Improvements in fine motor skills were also noted, possibly due to the physical interaction required during tablet gameplay. Parents highlighted that the program fostered positive social interactions among their child and other children, strengthened their child’s communication skills, and improved parent–child relationships by providing structured quality time. Parents made specific positive comments, as follows: “My child is completely changed; she is remembering things and can follow instructions,” “She is now able to socialize with friends without fighting as before,” “After malaria, she was hyperactive, but with this treatment, I see her becoming calmer and behaving more like herself,” and “The games have helped her school performance.” All parents who completed the study strongly agreed that they would participate in the program again, would highly recommend it to others, and desired to continue the intervention beyond the study’s completion.

Affordability was assessed at both the healthcare system and individual family levels. From the healthcare perspective, nurses reported minimal additional costs. The equipment packages proved durable, with all tablets functioning after 2 years, although one had a cracked screen. Solar panels experienced minor issues, with three of 10 requiring repairs, which were fixed locally at minimal cost. Six power banks were damaged, likely because of unexpected overuse within households. Despite these challenges, all the equipment was returned by families, with no losses or thefts, demonstrating the program’s cost-effectiveness. One hundred percent of families reported cost savings because they were able to use the solar system to charge their cellular devices, thus eliminating the need to pay to charge them at local shops.

Nurses reported that Brain Vital Signs was feasible and successfully integrated into existing workflows. It required minimal infrastructure, relying on charged laptops and the internet only for data uploads, which was beneficial in this resource-limited setting. Scans typically took ∼10 minutes, making the process time-efficient. Cultural and language barriers were minimal because the adapted use of Chichewa facilitated smooth communication with participants. The nurses reported that despite feeling fully supported, the time difference (9–10 hours) between them and the off-site team posed a challenge, as live assistance was unavailable during follow-up sessions.

The acceptability of Brain Vital Signs among families was high (100%). Brain Vital Signs also exhibited overall appropriateness, particularly for children aged 6 years and older. Younger children were more likely to fall asleep during scans, affecting data quality. Cap sizes were adequate, ensuring successful impedance on children of all ages in the study. External noise from the hospital ward occasionally disrupted data collection. The nurses expressed a desire to continue using Brain Vital Signs monitoring, recognizing its clinical utility in CM recovery monitoring.

## DISCUSSION

Cerebral malaria represents a significant health crisis in Africa, contributing heavily to long-term neurodisability, with few accessible neurorehabilitation solutions. In the present pilot study, the feasibility of two novel technologies was explored: one that delivers cognitive rehabilitation at home (DI) and one that quickly and objectively assesses brain functioning (Brain Vital Signs).

After the provision of tablets and solar charging devices, DI was feasibly implemented with a train-the-trainer model. Technical issues were quickly addressed with additional password protection and device training with families, resulting in no accounts being deleted after the 1-month follow-up. Despite making culturally appropriate adaptations, the core components of DI remained unchanged. Parent interviews indicated perceived cognitive and behavioral gains from the DI program. Nurses were enthusiastic that DI provided parents with an opportunity to work on their child’s cognitive function at home.

Literacy and language emerged as a significant barrier to acceptability. Future iterations should prioritize translating game instructions into the local language to minimize the need for external support, maximize the opportunity for impact, and decrease family and participant stress. Additionally, future iterations of DI should encourage equal playtime across all therapeutic games by locking overplayed games. Lastly, although the initial investment in equipment was not inexpensive ($950 per participant), the equipment exhibited good durability and could ideally be used to support the neurocognitive rehabilitation of many children. However, maintaining and troubleshooting equipment may incur additional costs, highlighting the need to evaluate financial feasibility over longer time periods. Additional costs were incurred in the present pilot study when nurses had to travel to remote villages to assist with troubleshooting devices or equipment. The authors of future studies could also work to leverage community support to reduce the need for nurse involvement after hospital discharge, potentially enhancing the program’s financial feasibility beyond research settings.

Brain Vital Signs monitoring was a feasible and acceptable way to assess brain health in this resource-limited setting, after key language adaptations were made. Although the cost of the required equipment exceeds that of the materials needed for standard cognitive testing, Brain Vital Signs are reusable. Nurses effectively delivered Brain Vital Signs after brief in-person training. The device integrated well into the workflow and patient care, although minor barriers, such as noise, were reported. Brain Vital Signs were easier to conduct with older children, whereas scans in younger children were more susceptible to inattention during testing. Overall, Brain Vital Signs shows promise as an accepted, appropriate, and scalable neuro assessment tool for this population and setting. The authors of future studies could evaluate the ability of all types of healthcare providers with various backgrounds to measure Brain Vital Signs, which may improve the generalizability of the approach.

### Limitations.

A limitation of the present study is the sample size, with only 19 children participating. Researchers conducting future program deployments to address the identified barriers should recruit a larger sample to assess the feasibility, acceptability, and cost-effectiveness of the program.

There were limitations to the authors’ ability to establish fidelity. Although the DI application includes gameplay time monitoring and internal fidelity checks, which sync back to the application’s internal servers, these data could not be reliably collected because of low bandwidth. As such, the research team relied on parental reports, manual tracking forms, and estimated times based on progress through game levels. The authors of future studies should ensure adequate bandwidth to collect and review internal gameplay data.

Because the present study was a feasibility study, a key limitation was reliance on subjective measures, such as parents’ perceptions of memory changes, to measure appropriateness, resulting in vulnerability to bias and halo effects. To determine if DI is clinically efficacious in this population, a larger randomized controlled trial that includes pre- and post-treatment Brain Vital Signs assessments is required. Additionally, the timeframe of the present study was limited to a 6-month follow-up, with anecdotal evidence revealing that benefits improve over time. Longer-term follow-up will be required in future trials to determine if cognitive improvements persist after DI completion. The authors of future research may also evaluate the efficacy of cognitive rehabilitation programs such as DI when considering underlying baseline immunopathogenic biomarkers, as differential recovery has been demonstrated on the basis of the degree of underlying neuroinflammation.^32^

## CONCLUSION

The present pilot study indicates that DI and Brain Vital Signs hold promise as neurorehabilitation and brain function-monitoring options for children with CM in remote communities. Among a small sample of participants, the protocol was found to be feasible and yielded high levels of acceptance and appropriateness, as reported by families and healthcare providers. Further research with a larger sample size, longitudinal data, and age-matched controls is warranted to support the efficacy of this program.
